# Optical coherence tomographic angiography study of perfusion recovery after surgical lowering of intraocular pressure

**DOI:** 10.1038/s41598-021-96225-7

**Published:** 2021-08-26

**Authors:** Liang Liu, Hana L. Takusagawa, Miles F. Greenwald, Jie Wang, Brock Alonzo, Beth Edmunds, John C. Morrison, Ou Tan, Yali Jia, David Huang

**Affiliations:** Casey Eye Institute and Department of Ophthalmology, Oregon Health and Science University, 515 SW Campus Drive, Portland, OR 97239-4197 USA

**Keywords:** Glaucoma, Imaging and sensing

## Abstract

We investigated the time and location of retinal perfusion recovery after surgical intraocular pressure (IOP) lowering in glaucoma by using optical coherent tomography angiography (OCTA). Seventeen patients were analyzed. The 4.5 × 4.5-mm OCTA scans centered on the disc were performed preoperatively and postoperatively at 6 weeks, 3 months, and 6 months. The peripapillary retinal nerve fiber layer (NFL) thickness, NFL plexus capillary density (NFLP-CD) and visual field (VF) were measured overall and in 8 corresponding sectors. The low-perfusion area (LPA) was used to assess the cumulative area where local NFLP-CD was significantly below normal. At 6 months, the average IOP decreased 5.3 mmHg (*P* = 0.004), LPA decreased by 15% (*P* = 0.005), and NFLP-CD improved by 12% (*P* < 0.001). The NFL thickness and VF mean deviation didn’t change significantly at any time point. Among the sectors with significant preoperative NFLP-CD loss, the recovery at 6 months was greatest in sectors with minimal preoperative NFL thinning (*P* < 0.001). In conclusion, surgical IOP lowering may improve NFLP capillary perfusion after 6 months. The perfusion recovery tended to occur in areas with minimal NFL thinning at baseline. OCTA parameters may have potential usefulness as pharmacodynamic biomarkers for glaucoma therapy.

## Introduction

Glaucoma is a leading cause of irreversible blindness in the world and a major source of disability and morbidity in the United States^1,2^. The current treatment of glaucoma has focused on reducing the intraocular pressure (IOP) to slow the progression of the disease. There is evidence that visual field (VF) function could improve after glaucoma surgery, suggesting that some retinal ganglion cells could recover their function if the IOP is sufficiently reduced^3,4^. However, such recovery is difficult to demonstrate by VF due to its poor reproducibility. Very large samples sizes and prolonged follow-up are required to demonstrate statistically significant improvement of VF defects or a decrease in progression^4–12^.

In the last 5 years, optical coherence tomographic angiography (OCTA) has emerged as a noninvasive imaging modality that can complement VF and structural OCT in glaucoma assessment. OCTA parameters such as vessel density and capillary density of the peripapillary retina and the macula have shown high glaucoma diagnostic accuracy and high correlation with VF parameters^13–18^. The measurement of OCTA parameters can be affected by signal strength and other image quality issues, such as shadow or vignetting artifacts, background bulk motion noise^19,20^. OCTA parameters show a diagnostic accuracy of detecting glaucoma comparable with structural OCT parameters in some studies^15–18^, whereas other studies demonstrated structural OCT parameters perform better^21,22^.

Recently, OCTA has been used to investigate responses to glaucoma treatments, with some studies showing improved retinal perfusion following IOP-lowering surgery^23–28^. This is of interest as retinal perfusion could be linked to ganglion cell metabolism and function. Our group has recently developed new methods for assessing focal perfusion changes in the nerve fiber layer plexus (NFLP), the plexus most affected by glaucoma in the peripapillary region^15,29,30^. In this study, we applied these new tools to investigate the extent, location, and timing of perfusion recovery following surgery.

## Results

### Study population

Twenty-two participants were enrolled. One participant was excluded due to postoperative hypotony and 4 participants were excluded due to poor image quality—leaving one eye each of 17 participants for statistical analysis (Table 1). Of these, 11 participants had mild glaucoma and 6 participants had moderate glaucoma according to the modified Hodapp–Parrish–Anderson classification system^31^. All were classified as primary open-angle glaucoma (POAG). All were using at least one ocular antihypertensive medication preoperatively. Trabeculectomy was performed in 15 participants and canaloplasty was performed in 2 participants by different surgeons.

**Table 1:** Participant characteristics.

Parameter	Preoperative	Postoperative (6 months)	*P* value
Participant count	17	Same	
Eye count	17	Same	
Age (years)	70 ± 6	Same	
Male/female count	6/11	Same	
Best corrected visual acuity (LogMAR)	− 0.02 ± 0.07	0.01 ± 0.08	0.219
Intraocular pressure (mm Hg)	15.9 ± 3.8	10.6 ± 2.7	**0.005**
Topical ocular antihypertensive, count	2.1 ± 1.1	0.2 ± 0.7	** < 0.001**
Systolic blood pressure (mm Hg)	128.1 ± 13.7	125.8 ± 15.4	0.782
Diastolic blood pressure (mm Hg)	82.8 ± 10.5	82.4 ± 11.2	0.899

*MD* mean deviation, *PSD* pattern standard deviation, *NFL* nerve fiber layer, *NFLP-CD* retina nerve fiber layer plexus capillary density.

Group means ± standard deviations are shown unless otherwise noted. The Wilcoxon signed rank test was used.

Statistically significant *P* values (< 0.05) are in bold type. For the count of topical anti-ocular hypertensives, combination agents are counted as 2 medications.

There was no statistically significant difference at preoperative and 6 months postoperative visits for best corrected visual acuity and systolic/diastolic blood pressures (Table 1). As expected, participants had significantly lower IOP and less ocular antihypertensive eye drops use 6 months postoperatively. IOP reduction ranged from 1 to 10 mm Hg.

### Outcome parameters at preoperative and postoperative visits

After surgery, IOP control significantly improved by 3 months (Table 2). Improvements in perfusion and anatomic parameters lagged—they were only significant at 6 months and not at the earlier visits. Both OCTA perfusion parameters were improved—LPA decreased by 15% and NFLP CD increased by 12%. Some disc anatomic parameters improved—the rim area increased by 8.8% and the cup/disc area ratio decreased by 9.3%. There were no significant correlations (*P* > 0.226) between the perfusion and anatomic improvements and the amount of surgical IOP reduction. There were no statistically significant changes in NFL thickness, VF parameters, or OCTA images signal strength index at any of the postoperative visits (Table 2). The median values and interquartile ranges of OCT and OCTA parameter were showed in Supplementary Table S1.

**Table 2:** Preoperative values and postoperative changes in outcome parameters.

Parameters	Preoperative	Postoperative–preoperative difference			*P* value
		6 weeks	3 months	6 months	
IOP (mmHg)	15.9 ± 3.8	− 3.7 ± 6.2	− **4.7 ± 4.3**	− **5.3 ± 3.3**	**0.001**
Low perfusion area (mm^2^)	3.91 ± 2.52	− 0.18 ± 0.54	− 0.003 ± 0.98	− **0.57 ± 0.72**	**0.006**
NFLP-CD (% area)	42.7 ± 15.8	2.4 ± 5.0	1.0 ± 6.1	**5.3 ± 6.0**	**0.001**
NFL thickness (μm)	67.6 ± 18.8	0.1 ± 3.4	− 1.4 ± 4.0	− 0.3 ± 4.0	0.486
Rim area (mm^2^)	1.02 ± 0.39	0.02 ± 0.07	0.03 ± 0.07	**0.09 ± 0.08**	**0.002**
Cup volume (mm^3^)	0.15 ± 0.17	0.00 ± 0.01	− 0.02 ± 0.04	− 0.02 ± 0.07	0.157
Cup/disc area ratio	0.43 ± 0.16	0.00 ± 0.05	− 0.01 ± 0.05	− **0.03 ± 0.04**	**0.003**
VF MD (dB)	− 4.61 ± 3.33	N/A	-0.22 ± 1.68	0.02 ± 1.27	0.779
VF PSD (dB)	6.3 ± 3.3	N/A	− 0.6 ± 0.8	− 0.1 ± 1.1	0.192
VF VFI (%)	88.5 ± 9.5	N/A	0.1 ± 3.3	0.6 ± 2.3	0.651
OCTA images signal strength index	63 ± 5	58 ± 7	61 ± 7	61 ± 6	0.145

*MD* mean deviation, *PSD* pattern standard deviation, *VFI* visual field index, *NFL* nerve fiber layer, *NFLP-CD* retina nerve fiber layer plexus capillary density, *OCTA* optical coherence tomography angiography.

Group means ± standard deviations are shown*. P* values are from the Friedman test to compare the preoperative values and the 3 postoperative values. Statistically significant differences between any one postoperative visit and the preoperative visit (*P* < 0.05/3, Wilcoxon test with Bonferroni correction for 3 postoperative visits) are shown in bold type.

### Localization of retinal perfusion improvement

**Figure 1:**
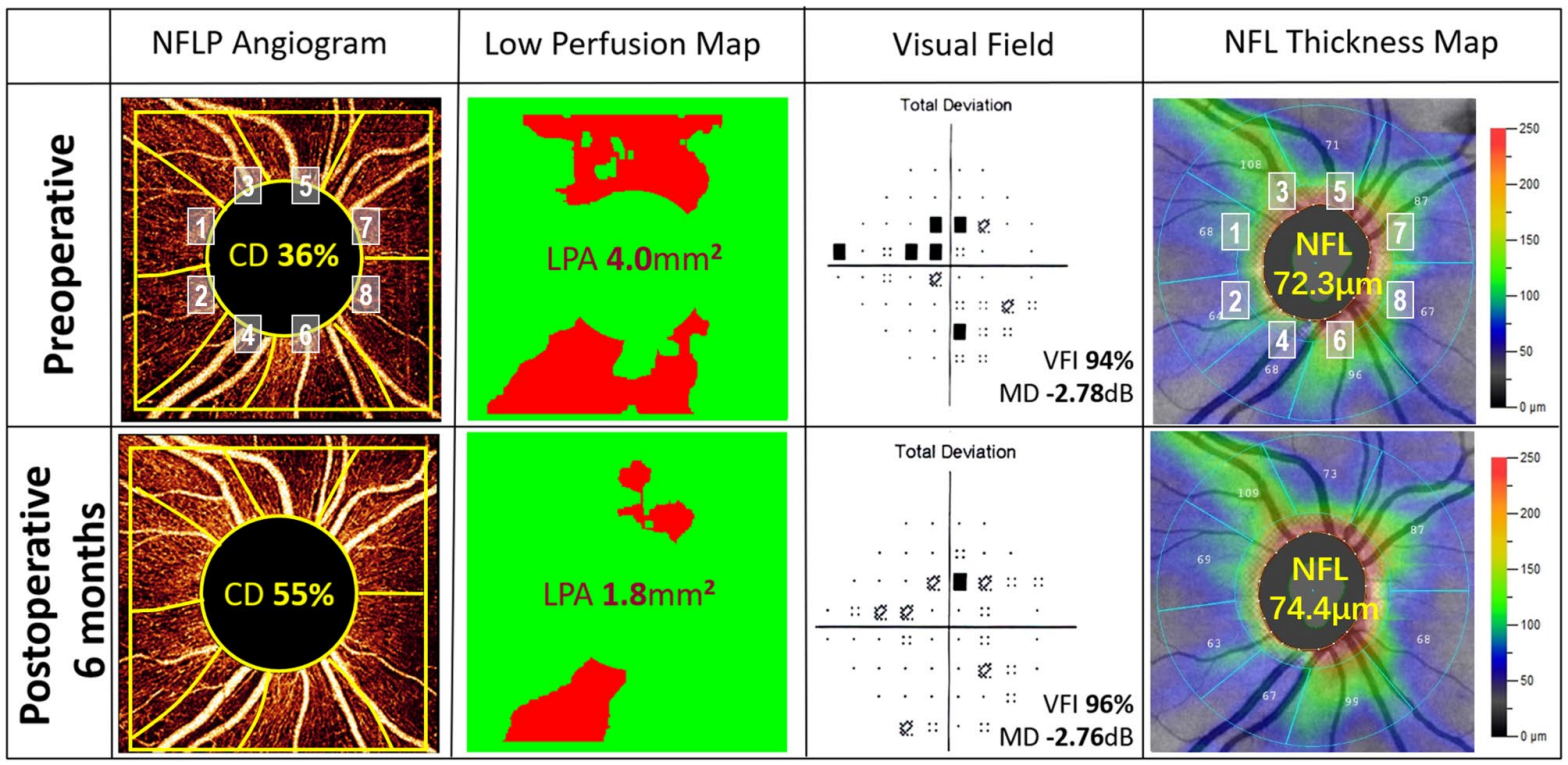
A mildly glaucomatous eye showed retinal perfusion recovery at 6 months after trabeculectomy. The nerve fiber layer plexus (NFLP) angiogram was divided into 8 corresponding sectors according to an extended Garway-Heath scheme. This participant had lower IOP (from 16 to 13 mmHg) and less ocular antihypertensive medications use (from 3 to 0) at 6 months postoperatively. The NFLP capillary density (CD) increased by 53%. The low-perfusion map showed the perfusion defects mainly in the sector 3, 4, 5 and 6. The low-perfusion area (LPA) became 55% smaller after surgery. The perfusion recovery occurred primarily in the sector 3 and sector 6, where the NFL thickness were less thinning than the sector 5 and sector 4. The visual field (VF) total deviation map showed shallower defects. The rim area increased from 1.10 to 1.21 mm^2^ and the cup/disc area ratio decreased from 0.30 to 0.27. However, the VF mean deviation (MD) and nerve fiber layer (NFL) thickness showed minimal improvement.

An eye with mild glaucoma was chosen to demonstrate retinal perfusion recovery 6-month postoperatively (Fig. 1). Wedge-shaped areas of capillary dropout in the superior and inferior hemispheres could be visualized in both the NFLP angiogram and low-perfusion map at the preoperative visit. The low-perfusion map showed the perfusion defects mainly in the sector 3, 4, 5 and 6. Postoperatively, there was more than 50% increase in the average NFLP-CD and reduction in the area of perfusion defect (LPA). The perfusion recovery occurred primarily in the sector 3 and sector 6, where the NFL thickness were less thinning than the sector 5 and sector 4. There were also anatomic and VF improvements, but they were minimal by comparison to the perfusion changes.

### Sectoral analysis of retinal perfusion improvement

**Figure 2:**
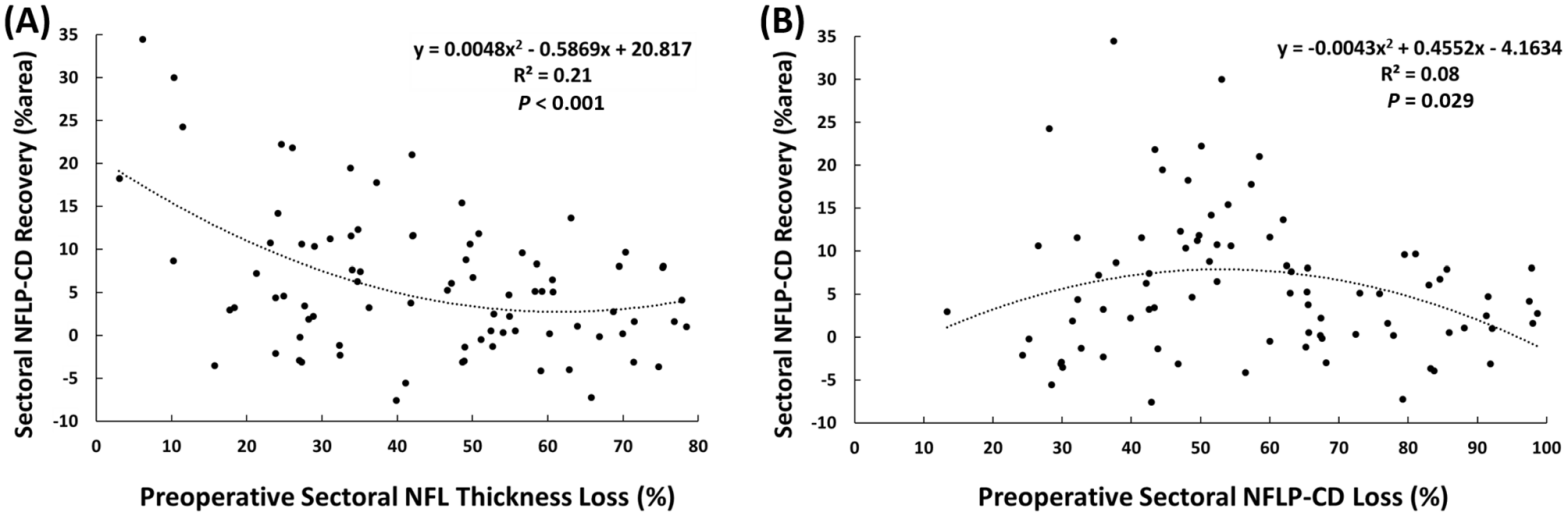
The recovery of nerve fiber layer plexus capillary density (NFLP-CD) 6 months after surgery was correlated with preoperative parameters on a sector basis. Sectors with significant preoperative NFLP-CD loss were analyzed. (**A**) The plot against preoperative nerve fiber layer (NFL) thickness loss showed that the perfusion recovery was greatest in sectors with minimal preoperative NFL thinning (P < 0.001). (**B**) Plot against preoperative NFLP-CD loss showed greater recovery was possible in sectors with moderate preoperative perfusion loss (P = 0.029).

The *en fa*ce OCTA of the NFLP was divided into 8 sectors according to a modified Garway-Heath scheme (Fig. 1). At the baseline visit, the NFLP-CD of all the 136 sectors was 42.4% area (median value, with the interquartile range from 27.9 to 60.7% area). At 6 months, there was a significant sectorwise perfusion recovery (P < 0.001, Wilcoxon test). The NFLP-CD improved to 53.2% area (median value, with the interquartile range from 32.8 to 63.9% area). Among the 8 modified Garway-Heath sectors in the 17 study eyes, there were 84 sectors (62%) with significant preoperative CD loss (more than 1.96 standard deviation below normal mean). In these 84 sectors, the average improvement of sectoral NFLP-CD at 6 months was 6.7% area (P = 0.003, linear mixed-effects model, with 95% confidence interval 2.3 to 11.1% area improvement). These sectors were pooled for regression analysis (Fig. 2). In univariate regression analyses (Fig. 2), the sectorwise perfusion recovery, as measured by the 6-month increase in NFLP-CD, was greatest in sectors with minimal preoperative NFL thinning (P < 0.001) (Fig. 2A). And the greater perfusion recovery was possible in sectors with moderate preoperative perfusion loss (P = 0.029) (Fig. 2B). The multivariate regression analysis yielded the following formula for predicting the postoperative NFLP-CD recovery (R^2^ = 0.38, *P* < 0.006):

$$NFLP-CD \,recovery=0.635\times {TLB}^{2}-0.801\times TLB-0.577\times {CDLB}^{2}+0.786\times CDLB+0.0306,$$

where $TLB$ is NFL thickness percent loss at baseline, $CDLB$ is NFLP-CD percent loss at baseline.

## Discussions

Our study agrees with 2 previous OCTA studies^24,25^ that showed improvement in peripapillary retinal perfusion after IOP-lowering surgery. These 2 studies had significant IOP reductions (18 mmHg and 16 mmHg, respectively) and eyes with more severe glaucoma (VF MD − 13 dB and − 16 dB, respectively), compared to our study. There is also evidence of a similar perfusion improvement after medical reduction of IOP^26^. Four OCTA studies had not found a significant difference in peripapillary retinal perfusion following IOP-lowering surgery^23,27,28,32^. These four studies had relatively small IOP reductions (from 7 to 10 mmHg) and eyes with moderate to severe glaucoma (average VF MD ranged from − 10 to − 15 dB). One of these study^27^ only looked at the 1-month postoperative outcome, which is too soon for perfusion improvement to occur, according to our results. Although the average reduction of IOP was only 5.3 mmHg in our study, we still detected a significant improvement of the peripapillary retinal perfusion. One possible explanation is our study had more mildly glaucomatous eyes with average VF MD − 4.6 dB. Since these eyes had less NFL thinning than severe glaucomatous eyes, they might have more perfusion recovery potential.

While the final outcome of this study is the same as previous publications, we were able to analyze the timing and location of perfusion improvements to obtain novel insights. The increase in NFLP-CD and reduction in LPA both occurred gradually after surgery, only becoming significant after 6 months. The perfusion recovery tended to occur in areas with minimal NFL thinning and moderate perfusion loss. These patterns are consistent with the hypothesis that in glaucomatous eyes there exist dysfunctional ganglion cells and nerve fibers that have reduced metabolism and perfusion. These cells have not yet died or lost volume and thickness. They can gradually recover function, metabolism, and perfusion after IOP is reduced to a tolerable level.

Since the perfusion recovery appears to be caused by the IOP reduction, there should be a correlation between the two. However, we did not find a significant correlation between the amount of IOP reduction and the amount of perfusion recovery. This may be due to the modest (mean 5, range 1–10 mm Hg) IOP reduction in our study. In a similar sized trabeculectomy study with a more dramatic IOP reduction (mean 18 mm Hg), In et al. did find that the perfusion recovery was significantly correlated to amount of the IOP reduction^24^.

The existence of injured but viable cells capable of recovery had already been proposed^33^ to explain the subset of patients with VF recovery after effective intervention^3,4,34,35^. Longitudinal data from the Collaborative Initial Glaucoma Treatment Study (CIGTS) showed that over 5 years, 14% of patients undergoing IOP-lowering therapy had significant improvement in their VF compared to baseline and that better IOP control was a predictive factor for VF improvement^3^. Caprioli et al. also reported improvement in VF 5 year following trabeculectomy^4^. However, others have found that VF loss did not significantly improve following IOP-lowering^5,6^. These findings can be reconciled if we take into account the high test–retest variability of VF results, which necessitates many tests and large cohorts to yield statistically significant results. The findings in our modest cohort suggest that OCTA may be able to detect post-intervention changes more quickly and easily than VF.

While viable ganglion cells could recover, cells that have undergone apoptosis should not be able to regenerate. Thinning of the NFL is an indication of cell loss, which theoretically would be irreversible. We found that NFL thickness did not change significantly after surgery. This agrees with previous studies, which showed either no change after IOP-lowering interventions^36^, inconsistent fluctuations^37^, or continued thinning^38^.

The ONH structure tells a more complicated story than the NFL. Several studies have documented increased ONH rim and reduced cupping after trabeculectomy^38–40^. We also found reduced cup-to-disc ratio and increased rim area. These changes, again, occurred gradually and lagged IOP reduction, suggesting that the phenomenon may be due to tissue remodeling rather than elastic rebound. The ONH changes were unlikely to be due to regeneration of nerve fibers, since NFL thickness did not increase after intervention. Therefore, the restoration of ONH rim area was likely due to the anterior migration of the scleral attachment of the lamina cribrosa—a reversal of the posterior migration that is known to occur in glaucoma^41^. Thus we do not believe that ONH structural recovery and NFLP perfusion recovery are causally related to each other in a direct way, although both are the sequelae of IOP reduction in glaucomatous eyes.

An exciting implication of our findings is that OCTA parameters could be used as pharmacodynamic biomarkers to assess the response to glaucoma treatments that are not based on lowering the IOP. Unlike ONH structural parameters, which measures IOP-mediated biomechanical changes and tissue remodeling, NFLP perfusion parameters respond to the metabolic demands of ganglion cells and nerve fibers. Thus if a neuroprotective agent could rescue viable but dysfunctional ganglion cells^42–44^, its effect could theoretically be measured by improvements in NFLP-CD or LPA. The excellent correlation between these OCTA parameters and VF parameters also support this possibility^15,45,46^. There is a great need for more precise objective biomarkers because VF parameters have high measurement variability^47,48^ and require large cohorts and many tests over extended follow-up periods to show significant improvement or slowing of progression.. Even the memantine study, with over 2000 patients and 48 months of follow-up, fell short of statistical significance in demonstrating the slowing of VF progression^42^. As our study shows, OCTA could demonstrate a highly significant treatment effect with only a small sample followed over a 6-month period, when VF parameters showed only small changes that were far from statistical significance. However, further studies are needed to verify that the short-term improvement in OCTA perfusion parameters could be linked to either long-term VF improvement or slow-down in progression.

Our results also suggest that OCT and OCTA parameters could be useful as predictive biomarkers—peripapillary areas with minimal NFL thinning and moderate NFLP-CD loss were most likely to recover. However, the level of agreement between predicted and actual recovery was only fair. Thus, its utility for individual patients may be limited. But these parameters could still be useful for the selection of patients most likely to respond to certain therapies on a group basis.

While we suggest many possible implications of our results, we realize that our study is limited to a short-term follow-up of a small sample of POAG patients that underwent IOP-lowering surgery. Some postoperative changes may be confounding. Stopping of glaucoma medications postoperatively may influence retinal perfusion, although published results so far only showed that medications have no change or improved capillary density^49,50^. Ocular surface changes may affect the quality of OCTA images, although our signal strength analysis showed that preoperative and postoperative images quality were comparable. Larger and longer studies are needed with more diverse glaucoma types and interventions.

In conclusion, OCTA showed that peripapillary NFLP perfusion gradually improved over 6 months after IOP-lowering surgery in glaucoma patients. The perfusion recovery tended to occur in areas with minimal NFL thinning and moderate perfusion loss. These findings suggest that OCTA parameters may have value in future studies to determine its potential usefulness as pharmacodynamic biomarkers for the recovery of dysfunctional but viable ganglion cells. They might be helpful in clinical trials of novel glaucoma treatments.

## Methods

### Study population

This prospective cohort study was performed from May 6, 2014 to February 11, 2016 at the Casey Eye Institute, Oregon Health & Science University (OHSU). The research protocols were approved by the Institutional Review Board at OHSU, and carried out in accordance with the tenets of the Declaration of Helsinki. Written informed consent was obtained from each participant.

The participants were part of the “Functional and Structural Optical Coherence Tomography for Glaucoma” study. The participants were enrolled at the Glaucoma Clinic of the Casey Eye Institute by the clinical investigators. All participants had progressive perimetric glaucoma (PG) and were scheduled to undergo trabeculectomy or canaloplasty surgery to lower intraocular pressure (IOP). The inclusion criteria were: (1) an optic disc rim defect (thinning or notching) or NFL defect visible on slit-lamp biomicroscopy; and (2) a consistent glaucomatous pattern, on both qualifying Humphrey SITA 24-2 VFs, meeting at least one of the following criteria: pattern standard deviation (PSD) outside normal limits (P < 0.05) or glaucoma hemifield test outside normal limits.

The exclusion criteria were: (1) best-corrected visual acuity (BCVA) worse than 20/40; (2) age < 30 or > 80 years; (3) refractive error of >  + 3.00 D or <  − 7.00 D; (4) previous intraocular surgery except for an uncomplicated cataract extraction with posterior chamber intraocular lens implantation; (5) any diseases that may cause VF loss or optic disc abnormalities; or (6) inability to perform reliably on automated VF testing. One eye from each participant was scanned and analyzed.

All ocular antihypertensive medications were continued up to the time of surgery. Eyes with signs of hypotony, maculopathy, or disc edema after surgery were excluded from analysis.

### Visual field testing

VF tests were performed with the Humphrey Field Analyzer II (Carl Zeiss, Inc.) set for the 24-2 threshold test, size III white stimulus, using the SITA standard algorithm. VF testing was done within 1 month prior to the surgery and 3 and 6 months after the surgery.

### Optical coherence tomography equipment

A 70-kHz, 840-nm wavelength spectral-domain OCT system (Avanti, Optovue Inc.) with the AngioVue OCTA software (Version 2016.2.0.35) was used.

### Image acquisition and processing

Pharmacologic pupil dilation was used for OCTA scanning to optimize image quality. The peripapillary retinal region was scanned using a 4.5 × 4.5-mm volumetric angiography scan centered on the optic disc. Each volume was comprised of 304 line-scan locations^20,21^. Two sets of scans were performed within one visit. The OCT angiogram with higher signal strength index (SSI) was used in the following analysis. The OCTA scan was done within 1 month prior to the surgery for the glaucoma patients and 6 weeks, 3 months and 6 months after the surgery.

The merged volumetric angiograms were then exported for custom processing using the Center for Ophthalmic Optics & Lasers-Angiography Reading Toolkit (COOL-ART) software^51^. The OCTA scans contained both volumetric flow (decorrelation) data as well as structural (reflectance) data. Segmentation of the retinal layers was performed by automated MATLAB programs that operate on the structural OCT data. Further manual correction of the segmentation was conducted by certified graders, if required. An en face angiogram of retinal nerve fiber layer plexus (NFLP) was obtained by maximum flow (decorrelation value) projection. The NFLP slab was bounded by the inner limiting membrane and the outer edge of the nerve fiber layer.

### OCT angiography measurements

In order to achieve good alignment between preoperative and postoperative OCTA images, the analytic area was cropped to 4 × 4-mm from 4.5 × 4.5-mm OCTA scan. The analytic area was manually centered on the optic disc based on the en face reflectance image excluding the central 2 mm diameter circle. The NFLP capillary density (NFLP-CD) was defined as the percentage area occupied by capillaries. Arterioles and venules (larger vessels) were automatically identified by thresholding the en face mean projection of OCT reflectance signal within the retinal slab. After these larger vessels were excluded, the remaining angiogram was used to compute capillary density. A reflectance-adjustment algorithm was used to correct the artifactually lower flow signal in regions of reduced reflectance (e.g. due to media opacity or pupil vignetting)^52^. Previous clinical validation has shown this algorithm was able to remove the dependence of retinal OCTA measurements on the signal strength index and reduce population variation^45,52^. A scan was considered to be grossly decentered if the 4 × 4-mm analytic area was cropped by more than 5%. Grossly decentered scans were excluded.

The *en face* OCTA of the NFLP and the 24-2 VF total deviation map were divided into 8 corresponding sectors according to an extended Garway-Heath scheme (Fig. 1). The original Garway-Heath scheme divided the disc rim into 6 sectors^53,54^. We added horizontal dividing lines to the original nasal and temporal sectors to increase the total number of sectors to 8^29^. The sector boundaries were extended outward along nerve fiber trajectories obtained from structural OCT nerve fiber flux analysis^55^. The sectoral NFLP-CD and NFL thickness were converted to percent loss, ranging from 0 to 100%, according to the equation:

$$ \% {\text{ Loss }} = { 1}00 \times \left( {{\text{N }} - {\text{ value}}} \right)/{\text{N,}} $$

where N is the average value in age-matched normal subjects in the FS-OCT study^17^.

Another OCTA outcome measure was the low-perfusion area (LPA). The low-perfusion map and LPA assessed the location and severity of focal glaucoma damage with good agreement with VF^29^. Low perfusion was defined as CD below the 0.5 percentile normative threshold in a contiguous area of at least 0.053 mm^2^ (98.5 normative cutoff). The low-perfusion map displayed normal perfusion in green and abnormally low perfusion in red (Fig. 1). The LPA in each eye was defined by the cumulative area of pixels that met the low perfusion criteria.

Image quality was assessed for all OCTA scans. Poor quality scans with signal strength index (SSI) below 50, or registered image sets with residual motion artifacts (discontinuous vessel pattern) were excluded from analysis.

### Structural OCT measurements

All the structural OCT parameters were measured from OCTA scans. The retinal NFL thickness was measured along 3.45-mm diameter annulus of 0.1-mm width centered on the optic disc.

The optic nerve head (ONH) rim area, cup volume and cup/disc area ratio were calculated using AngioAnalytics (version 2018.1.0.22, Optovue Inc, Fremont, CA). The optic disc boundary was defined by the Bruch’s membrane opening. The Bruch’s membrane opening plane was used as the reference plane to separate the neuroretinal rim from the cup.

### Statistical analysis

The Friedman test, which is a nonparametric alternative for repeated-measures analysis of variance, was used to analyze the differences across multiple visits. Wilcoxon signed-rank test was used to compare between two visits. Spearman correlation was used to determine the relationships between the changes of OCT/OCTA parameters with the changes of IOP and VF parameters. In order to investigate the correlations between preoperative OCT/OCTA parameters and postoperative perfusion recovery potential, univariate and multivariate regression analyses were used. A linear mixed-effects model was used to account for within-subject correlation in sectoral analysis; the subject identity was treated as a random effect. All statistical analyses were performed with SPSS 20.0 (SPSS Inc., Chicago, IL) and MedCalc 10.1.3.0 (MedCalc Software, Ostend, Belgium, www.medcalc.be). The statistical significance was assumed at P < 0.05.

## Supplementary Information

Supplementary Information.
