# Supplementary material for: Optical coherence tomographic angiography study of perfusion recovery after surgical lowering of intraocular pressure
Source: Sci Rep. 2021 Aug 26;11:17251. doi: 10.1038/s41598-021-96225-7 (PMC8390670; doi:10.1038/s41598-021-96225-7)
Supplement: Supplementary file 1 — Supplementary Information. [file 41598_2021_96225_MOESM1_ESM.docx]

| Parameters | Preoperative | Postoperative–Preoperative Difference | | | *P* value |
| --- | --- | --- | --- | --- | --- |
|  |  | 6 weeks | 3 months | 6 months |  |
| Low Perfusion Area (mm^2^) | 3.48  2.17 to 4.60 | 2.96  2.11 to 4.36 | 3.62  2.09 to 4.05 | **2.05**  **1.53 to 4.03** | **0.006** |
| NFLP-CD (% area) | 41.4  36.2 to 55.1 | 47.7  0.392 to 0.516 | 44.6  38.8 to 54.1 | **51.2**  **40.9 to 60.6** | **0.001** |
| NFL Thickness (μm) | 66.7  53.4 to 76.9 | 67.7  50.1 to 80.4 | 65.2  51.8 to 78.2 | 67.9  51.3 to 83.2 | 0.486 |
| Rim Area (mm^2^) | 1.11,  0.82 to 1.25 | 1.18,  0.81 to 1.37 | 1.18,  0.80 to 1.31 | **1.23,**  **0.86 to 1.33** | **0.002** |
| Cup Volume (mm^3^) | 0.10,  0.07 to 0.16 | 0.10,  0.06 to 0.16 | 0.10,  0.07 to 0.15 | 0.08,  0.06 to 0.15 | 0.157 |
| Cup/Disc Area Ratio | 0.37,  0.30 to 0.50 | 0.38,  0.30 to 0.49 | 0.37,  0.29 to 0.48 | **0.36,**  **0.27 to 0.46** | **0.003** |
| OCTA Images  Signal Strength Index | 63  60 to 63 | 59  52 to 63 | 62  59 to 65 | 60  58 to 65 | 0.145 |

**Supplemental Table 1. Preoperative Values and Postoperative Changes in Outcome Parameters Shown as Group Medians and interquartile ranges**

Group medians and interquartile ranges are shown*. P* values are from the Friedman test to compare the preoperative values and the 3 postoperative values. Statistically significant differences between any one postoperative visit and the preoperative visit (*P* < 0.05/3, Wilcoxon test with Bonferroni correction for 3 postoperative visits) are shown in bold type. MD = mean deviation; PSD = pattern standard deviation; VFI = visual field index; NFL = nerve fiber layer; NFLP-CD = retina nerve fiber layer plexus capillary density; OCTA = optical coherence tomography angiography
